# The between Now and Then of Lung Cancer Chemotherapy and Immunotherapy

**DOI:** 10.3390/ijms18071374

**Published:** 2017-06-27

**Authors:** Roberta Visconti, Francesco Morra, Gianluca Guggino, Angela Celetti

**Affiliations:** 1 Institute for the Experimental Endocrinology and Oncology “G. Salvatore”, Italian National Council of Research, via S. Pansini 5, 80131 Napoli, Italy; francesco.morra@unina.it; 2 Thoracic Surgery Unit, Antonio Cardarelli Hospital, via A. Cardarelli 9, 80131 Napoli, Italy; gianlucaguggino@gmail.com

**Keywords:** lung neoplasms, antineoplastic agents, biomarkers, poly(ADP-ribose) polymerase inhibitors, CCDC6

## Abstract

Lung cancer is the most common cancer worldwide. Disappointingly, despite great effort in encouraging screening or, at least, a close surveillance of high-risk individuals, most of lung cancers are diagnosed when already surgically unresectable because of local advancement or metastasis. In these cases, the treatment of choice is chemotherapy, alone or in combination with radiotherapy. Here, we will briefly review the most successful and recent advances in the identification of novel lung cancer genetic lesions and in the development of new drugs specifically targeting them. However, lung cancer is still the leading cause of cancer-related mortality also because, despite impressive initial responses, the patients often develop resistance to novel target therapies after a few months of treatment. Thus, it is literally vital to continue the search for new therapeutic options. So, here, on the basis of our recent findings on the role of the tumor suppressor CCDC6 protein in lung tumorigenesis, we will also discuss novel therapeutic approaches we envision for lung cancer.

## 1. Introduction

Lung cancer is the most common cancer worldwide and its incidence, while decreasing in some countries—such as the United States—is expected to further rise in others—such as China—where there has been a dramatic increase in smoking during the past decades [1]. When feasible, surgical resection remains the treatment of choice for early stage, localized tumors and can be curative [2]. After surgical resection, adjuvant chemotherapy is prescribed to lower the risk of recurrence in patients with lymph node involvement [3,4]. However, despite great effort in encouraging smoking cessation, screening in high risk individuals, and prompt diagnostic procedures in symptomatic patients, most lung cancers are discovered when already locally advanced. In this case, in appropriately selected patients, neoadjuvant chemoradiotherapy can be used to reduce the tumor mass and achieve surgical resectability [3,5]. Otherwise, patients classified as having unresectable, locally advanced lung cancer are treated with concurrent chemoradiotherapy [3]. Chemotherapy is also the only palliative, systemic treatment for metastatic tumors [3]. Finally, chemotherapy is the treatment of choice for patients that, regardless of tumor stage, are not eligible for lung resection because of their respiratory and/or general status [3]. Thus, overall, more than 80% of newly diagnosed lung cancer patients receive chemotherapy, alone or in combination with radiotherapy. 

Despite recent great advances in the identification of novel targetable genetic lesions and in the development of new drugs, lung cancer is still the leading cause of cancer-related mortality: thus, one of the major human killers with a disappointing five-year survival rate of 15% [1]. It is, therefore, literally vital to search for new drugs or to improve the current treatment protocols to increase efficacy, overcome resistance, and reduce side effects. Here we will briefly review the most successful and recent advances in the therapy of lung cancer, especially of non-small-cell lung cancers (NSCLCs). Moreover, we will discuss new therapeutic options we suggest on the basis of our recent findings on the role of the tumor suppressor CCDC6 protein in lung cancer.

## 2. Discussion

### 2.1. Latest FDA-Approved Therapeutics for NSCLCs

NSCLCs, that mainly comprise adenocarcinomas and squamous cell carcinomas (SCCs), account for more than 85% of all lung cancers [6]. The combination of carboplatin and paclitaxel has long been the most common first-line therapeutic regimen prescribed for NSCLCs; however, in the last few years, NSCLC treatment has been dramatically changed by the FDA approval of many new target therapies (Table 1). 

About 5% of NSCLCs express the EML4-ALK fusion oncogenic protein in which the ALK kinase is constitutively active [17]. For ALK-positive NSCLC metastatic patients, the FDA has approved crizotinib—a small tyrosine kinase inhibitor—as the first line of therapy [7,8]. Last year, the FDA expanded crizotinib use also to the 1–2% of NSCLC patients with ROS1 rearrangements [8,9,18]. In both cases, however, despite an impressive initial response, patients develop a resistance after a few months of treatment. For ALK positive patients, which relapse after crizotinib treatment, there are three second-generation ALK inhibitors available: ceritinib, alectinib, and the very recently approved brigatinib [8,12,13,14].

Most of the recent FDA approved drugs for NSCLC therapy target the Epidermal Growth Factor (EGF) pathway. Afatinib is a second-generation irreversible inhibitor of both EGF receptor (EGFR) and epidermal growth factor receptor 2 (HER2). It is FDA-approved as frontline therapy in metastatic NSCLC patients with documented activating EGFR mutations and it is now preferred to the first-generation EGFR inhibitors erlotinib and gefinitib, as their long-term efficacy is limited by development of resistance [8,10]. The most common resistance mechanism is the development of a secondary mutation in EGFR, T790M [19]. Recently, after a very successful clinical trial, the FDA has approved osimertinib—a third generation selective EGFR T790M inhibitor—for patients that have progressed after therapy with first or second generation EGFR inhibitors [8,15]. Of note, in contrast to what established for advanced colorectal cancer, in NSCLC, at present, the evidence that K-Ras mutations predict a lack of benefit from EGFR-targeting therapy is not strong enough to impose K-Ras status investigation before starting treatment [20].

Another target in NSCLC therapy is the tumor angiogenetic Vascular Endothelial Growth Factor (VEGF) pathway. Ramucirumab, a monoclonal antibody that works as a receptor antagonist blocking the binding of VEGF to VEGFR2, is approved as second-line therapy in combination with docetaxel for EGFR- and ALK-negative patients with disease progression on or after platinum-based chemotherapy [8,16]. The monoclonal antibody bevacizumab, that binds to soluble VEGF preventing receptor binding, has been approved, in combination with platinum-based chemotherapy, for first-line treatment of unresectable, locally advanced, recurrent, or metastatic non-squamous NSCLCs [8,11]. The SCCs are excluded because of a higher risk of serious, life-threating hemorrhagic adverse events [21]. Moreover, unlike adenocarcinomas, lung SCCs rarely harbor EGFR and ALK mutations, thus, until very recently, there has not been significant improvement in their treatment [22]. Now, however, immunotherapies targeting the programmed cell-death-1 receptor (PD-1) or its ligand, PD-L1, have expanded the treatment options (Table 2). Physiologically, PD-1 is expressed on regulatory and cytotoxic activated T cells. Binding of PD-L1 to its receptor inactivates the T cells, a key mechanism to limit immune responses [23]. PD-1 is highly expressed on many tumor-infiltrating lymphocytes but cancer cells often overexpress PD-L1, so blocking the immune attack against themselves. This has provided a strong rationale for the development of drugs targeting the PD-1 pathway. Indeed, drugs blocking the binding of PD-L1 to its receptor, such as nivolumab and pembrolizumab, enhance immunity against a wide variety of cancers, including NSCLC [24]. Nivolumab was initially FDA-approved strictly for lung SCCs and regardless of PD-L1 expression analysis, thus becoming the second-line treatment of choice after failure of first-line platinum-based therapeutic regimes: a major breakthrough in the field of SCC therapy after years of quiet [8,25]. Later on, nivolumab has been approved also for lung adenocarcinoma patients with progression on or after platinum-based chemotherapy; however, patients with EGFR or ALK-1 genomic aberrations should also have had disease progression on FDA-approved therapy for these aberrations prior to receiving nivolumab [8,26]. Pembrolizumab is indicated for the first-line treatment of any histological type of metastatic NSCLC if the tumor is highly positive for PD-L1 expression and negative for EGFR and ALK-1 genetic aberrations [8,27,28]. In the second-line, pembrolizumab is indicated for the treatment of even low PD-L1 expressing NSCLCs that have progressed on or after platinum-based chemotherapy. However, again, patients with EGFR or ALK-1 genomic aberrations should have shown disease progression on FDA-approved therapy for these aberrations prior to receiving pembrolizumab [8]. Atezolizumab, another PD-L1 blocking antibody, has been approved for the treatment of patients with metastatic NSCLC whose disease progressed during or following platinum-containing chemotherapy. Also in this case, patients with EGFR or ALK genomic tumor mutations should have had disease progression on FDA-approved therapy for these aberrations prior to receiving atezolizumab [8,29]. Of note, until now, nivolumab, pembrolizumab, and atezolizumab have received FDA approval as single agents. Many clinical trials are ongoing to address the anti-PD-L1/PD-1 therapy efficacy in combination with other therapies and the results are eagerly awaited [30].

### 2.2. New Therapeutic Perspectives for Lung Cancers

As reviewed above, in the very last few years, several new drugs have been approved for the treatment of NSCLCs. The bad news is that we are now witnessing that, after dramatic initial responses, the long-term effects of these drugs are almost always hampered by complications, such as hyperprogression upon anti-PD-L1/PD-1 therapy, or by acquired resistance [31,32,33]. The good news is that we have learnt that the time gap between bench to bedside is getting shorter, so the search for novel genetic aberrations that can be exploited to further personalize therapy warrants investigation. 

We have recently demonstrated that in about 30% of NSCLC the tumor suppressor protein CCDC6 is expressed at low levels. Remarkably, CCDC6 low levels indicate poor prognosis, correlating positively with the presence of lymph node metastasis and negatively with disease free survival [34]. CCDC6, a negative regulator of CREB1 transcriptional activity and a substrate of ATM, sustains DNA damage checkpoints in response to genotoxic events, CCDC6 loss affecting the repair of the DNA double-strand breaks (DSBs) [35,36,37,38,39]. Accordingly, lung cancer cells expressing low levels of CCDC6 have severe defects in the homologous recombination (HR) DNA repair pathway induced by DSBs [34]. For the treatment of ovarian cancers, bearing HR DNA repair defects because of BRCA1/2 mutations, the FDA has approved olaparib, an inhibitor of the repair enzyme Poly(ADP-ribose) polymerase (PARP) [8,40,41]. Inhibition of PARP causes a collapse in the base excision repair (BER) pathway and results in the accumulation of single strand breaks that end up in DSBs upon DNA replication. In healthy cells, PARP inhibition is not of great consequence because of effective DSB repair. However, in the context of BRCA-mutated cancers with compromised HR repair, the BER pathway failure and, in turn, the accumulations of DSBs caused by the PARP inhibitor kill tumor cells [42]. Thus, given the defects in HR repair caused by CCDC6 loss, we hypothesized that CCDC6 defective lung cancer cells should have been sensitive to treatment with olaparib. Indeed, we have demonstrated that low levels of CCDC6 protein increase lung cancer cells’ sensitivity to the sole olaparib treatment; moreover, olaparib synergizes with cisplatinum in killing cells [34]. Thus, our data suggest that, in NSCLCs, expressing low levels of CCDC6 the addition of olaparib to first-line platinum-based chemotherapy can be beneficial. However, as discussed above, CCDC6 protein levels are downregulated in 30% of NSCLCs. For the tumors that express normal levels of the protein, we propose a different therapeutic approach. CCDC6 protein levels are finely regulated by the E3 ubiquitin ligase Fbxw7, that addresses CCDC6 to proteasome degradation, and by the de-ubiquitinase Usp7 that, on the contrary, stabilizes it [43]. Thus, we reasoned that P5091, an Usp7 inhibitor, lowering CCDC6 levels, should sensitize even lung cancer cells that express normal level of the protein to olaparib. Indeed, this is the case. Remarkably, P5091 synergizes with olaparib and cisplatinum also in cells derived from neuroendocrine small-cell lung cancers (SCLCs) [44]. These results suggest a new therapeutic option also for SCLCs that have been excluded by the recent advances in target therapies and are, thus, still treated only with platinum-based chemotherapy, with quite dismal results. 

CCDC6 was first identified as a gene frequently rearranged with the RET tyrosine kinase gene in papillary thyroid cancers [45]. In detail, as a consequence of the chromosomal rearrangement, the first 101 amino acids of the CCDC6 protein are fused to the RET tyrosine kinase domain that results constitutively active [46]. Albeit at low frequency, CCDC6 has been found rearranged with the tyrosine kinase RET also in NSCLCs [47]. In a phase II clinical trial vandetanib, a novel tyrosine kinase inhibitor of RET, showed clinical antitumor activity and a manageable safety profile in patients with advanced RET-rearranged NSCLCs [48]. In cells bearing CCDC6/RET rearrangements, CCDC6 function is completely lost because the CCDC6/RET chimeric protein acts as a dominant negative on the wild type CCDC6 protein codified by the non-rearranged allele [46]. Thus, the hypothesis that the functional loss of CCDC6 should sensitize also the RET-rearranged NSCLCs to olaparib—alone or in combination with vandetanib—is a promising starting point for further tests in preclinical and clinical settings.

## 3. Conclusions

In the last few years, many new target drugs have been approved by the FDA for the treatment of NSCLCs. Drugs targeting ALK, ROS, and the EGF and VEGF pathways have been particularly beneficial for patients with non-squamous NSCLCs. The very recent approval of drugs targeting the PD-L1/PD-1 pathway has been a groundbreaking advancement especially for the treatment of squamous NSCLCs. However, we have learnt that—after dramatic initial responses—the long-term effects of the target cancer therapies are limited mainly by acquired resistance. So, searching for new therapeutic options always warrants efforts. On the basis of the results obtained in NSCLC and SCLC-derived cell lines and in tumor samples, we envision CCDC6 as a biomarker for a more personalized lung cancer therapy. If the tumor expresses low levels of the protein, we expect it to respond to olaparib; if it expresses high levels of the protein the sensitivity to olaparib can be restored by the addition of the Usp7 inhibitor. Olaparib administration can be tested alone or in combination with classical chemotherapeutical drugs, such as cisplatinum, or with new target drugs, such as vandetanib in cases of CCDC6/RET-rearranged NSCLCs where the function of CCDC6 is lost. 

## Figures and Tables

**Table 1:** Target therapy for NSCLCs.

Target Protein	FDA Approved Drug	Relevant Clinical Trial Results
First Line Therapy		
ALK-1 ROS1	Crizotinib [7,8,9]	In ALK-1 positive tumors, PFS was significantly longer with crizotinib than with chemotherapy (median 10.9 Mos vs. 7.0 Mos; HR for progression or death with crizotinib, 0.45; 95% CI, 0.35–0.60; *p* < 0.001). In ROS1 positive tumors, ORR was 72% (95% CI, 58–84), with 3 complete and 33 partial responses. Median duration of response was 17.6 Mos (95% CI, 14.5-NR).
EGFR HER2	Afatinib [8,10]	Median OS 28.2 Mos (95% CI, 24.6–33.6) in the afatinib group and 28.2 Mos (20.7–33.2) in the pemetrexed-cisplatin group (HR 0.88; 95% CI, 0.66–1.17; *p* = 0.39). Median OS 23.1 Mos (95% CI, 20.4–27.3) in the afatinib group and 23.5 Mos (18.0–25.6) in the gemcitabine-cisplatin group (HR 0.93; 95% CI, 0.72–1.22; *p* = 0.61).
VEGF	Bevacizumab [8,11]	Compared with chemotherapy alone, bevacizumab significantly prolonged OS (HR 0.90; 95% CI, 0.81–0.99; *p* = 0.03), and PFS (0.72; 95% CI, 0.66–0.79; *p* < 0.001).
Second Line Therapy		
ALK-1	Ceritinib [8,12] Alectinib [8,13] Brigatinib [8,14]	For ceritinib, median PFS was 18.4 Mos (95% CI, 11.1-non-estimable) in ALK inhibitor-naive patients and 6.9 Mos in ALK inhibitor-pretreated patients. For alectinib, median PFS was 8.9 Mos (95% CI, 5.6–11.3). For brigatinib, median PFS was 9.2 Mos (95% CI, 7.4–15.6) and 12.9 Mos (95% CI, 11.1-NR) depending on the dosage.
EGFR (T790M)	Osimertinib [8,15]	Median PFS was 9.6 Mos (95% CI, 8.3-NR) in EGFR T790M-positive patients and 2.8 Mos (95% CI, 2.1–4.3) in EGFR T790M-negative patients.
VEGFR2	Ramucirumab [8,16]	Median PFS was 4.5 Mos for the ramucirumab group compared with 3.0 Mos for the control group (*p* < 0.0001).

Abbreviations: PFS = progression free survival; Mos = months; HR = hazard ratio; CI = confidence interval; ORR = objective response rate; NR = not reached; OS = overall survival.

**Table 2:** Immunotherapy for NSCLCs.

Tumor Characteristics		FDA Approved Drug
High PD-L1 expression Absence of EGFR or ALK-1 aberrations	First Line Therapy	Pembrolizumab [8,27,28]
Low PD-L1 expression Disease progression on FDA-approved therapy	Second Line Therapy	Pembrolizumab [8,27,28]
Disease progression on FDA-approved therapy	Second Line Therapy	Nivolumab [8,25,26] Atezolizumab [8,29]

## Abbreviations

CCDC6	Coiled Coil Domain Containing protein 6
NSCLC	Non-Small-Cell Lung Cancer
FDA	Food and Drug Administration
SCC	Squamous Cell Carcinoma
EGFR	Epidermal Growth Factor Receptor
VEGF	Vascular Endothelial Growth Factor
PD-1	Programmed cell-Death-1 receptor
PD-L1	Programmed cell Death-Ligand 1
DSB	Double-Strand Break
HR	Homologous Recombination
PARP	Poly(ADP-Ribose) Polymerase
BER	Base Excision Repair
SCLC	Small-Cell Lung Cancer
